# Human biologic monitoring based on blood donations to the National Blood Services

**DOI:** 10.1186/s12889-020-08588-7

**Published:** 2020-04-08

**Authors:** Lior Hassan, Asher Moser, Efrat Rorman, Luda Groisman, Yamit Naor, Eilat Shinar, Roni Gat, Eli Jaffe, Victor Novack, Itai Kloog, Lena Novack

**Affiliations:** 1 Faculty of Health Sciences, Ben-Gurion University of the Negev, Sderot Rager 151, 84101 Beer-Sheva, Israel; 2 Negev Environmental Health Research Institute, Soroka University Medical Center, Beer-Sheva, Israel; 3 Blood Services Center, Magen David Adom, Tel Aviv-Yafo, Israel; 4 National Public Health Laboratory, Tel Aviv-Yafo, Israel; 5 Soroka University Medical Center, Beer-Sheva, Israel; 6 Department of Geography, Ben-Gurion University of the Negev, Beer-Sheva, Israel

## Abstract

**Background:**

The ambient exposure does not always reflect the internal levels of pollution absorbed in the body. While human biomonitoring (HBM) could provide a valid estimate of exposure extent, it is usually an expensive and a heavily manpowered enterprise. Using samples collected during blood donations for HMB may provide a more efficient platform for a routine biomonitoring.

**Methods:**

The current study is aimed to explore the feasibility of using the national blood banking system for the purposes of HBM, to compare between residents of a suspected polluted area in northern Israel (Haifa Bay) to the rest of the country. Specifically, we will assemble a geographically representative sample of blood donors residing in the study area and of the general population, to test for four industry and traffic-related metals: lead (Pb), cadmium (Cd), arsenic (As) and chromium (Cr). Samples of whole blood from donors will be tested in the Laboratory of Public Health Services managed by the Ministry of Health. The information on donors’ biomarkers levels will be further linked with the air pollution and meteorological data assessed at the location of the blood collection sites (short-term exposure) and donors’ permanent address (long-term exposure), as recorded by the monitoring stations spread throughout Israel and the satellite-based exposure models. The association between biomarkers and ambient environmental exposures will be assessed.

The samples’ collection is planned for 2 years of 2020–2021.

**Discussion:**

The information collected in this study could lead to environmental regulations within Haifa Bay area aimed to prevent exposure to high levels of hazardous chemicals.

## Background

An exposure level in vivo can be measured using a human biomonitoring (HBM) methodology, providing a personal level of exposure. Pollutants’ levels verified by HBM are instrumental for assessing, controlling and providing the appropriate guidelines to minimize the exposures to potentially harmful environmental chemicals [1].

HBM studies have been historically used in the field of occupational health. However, its focus has changed in recent years towards environmental exposure in the general population. For instance, an HBM of environmental chemicals in the Canadian Health Measures Survey is a comprehensive initiative providing general population HBM data in Canada. It is an ongoing, cross-sectional, direct measures survey implemented in 2-year cycles, with up to 7000 people in each cycle. Its recent 2016 report has covered information on biomonitoring results for 279 chemicals and other trace elements such as polychlorinated biphenyls (PCBs), organochlorines, flame retardants, perfluoroalkyl substances, volatile organic compounds (VOCs) and metabolites, environmental phenols, triclocarban, acrylamide, pesticides (e.g., triazines, carbamates, organophosphates, phenoxy, pyrethroids) and/or their metabolites, chlorophenols, polycyclic aromatic hydrocarbon (PAH) metabolites, phthalates and alternate plasticizer metabolites, and tobacco biomarkers [2].

The Center of Disease Control and Prevention (CDC) has been providing similar information on the nationally representative biomonitoring data in the general population in the USA since 1999. The analyses of blood, serum, and urine samples from random subsamples of 2-year surveys (National Health and Nutrition Survey - NHANES), typically include up to 6000–7000 participants at each round and an extensive list of chemicals tested, comparable with the Canadian survey [3].

HBM projects have been carried out in European countries, as well. The Environmental Health Monitoring System (EHMS) in the Czech Republic, which was established in 1994 has been conducting a series of annual HBM surveys to examine exposure to heavy metals and PCBs in a large number of biologic tissues [4].

Another research group from Havre Cedex, France, have published two reports on the “Metallic Profile” of whole blood in plasma in healthy adults in 2013 [5] and healthy children in 2015 [6]. Both samples were relatively small, with 106 and 99 volunteers, respectively.

The Israeli Ministry of Health conducted two biomonitoring studies. The first was held among 250 adults in 2011 [7–11] and assessed exposure to bisphenol A, organophosphate pesticides, phthalates, cotinine, polycyclic aromatic hydrocarbons, and the phytoestrogenic compounds genistein, and daidzein, based on analysis of urine samples. The second study few years later (2015–2016) and was integrated in the Ministry of Health National Health and Nutrition Survey (MABAT) [12]. Urine samples were analyzed for pesticides and cotinine in a sub-sample of 200 adults and 100 children [13].

HBM projects are usually expensive, labor intensive and, therefore, result in low-density sampling of the population, both spatially and temporally. Existing format of relatively small-sized bio-monitored cohorts preclude from drawing robust conclusions to research questions. For instance, to compare the degree of pollution exposure around an industrially active area to the rest of Israel, we would need a geographically representative sample of residents of the country that could not be provided by a relatively small national survey (the two last including 200–250 subjects). In case we are interested to investigate a certain event resulting in elevated pollution – we would not have sufficient number of subjects tested at the precise time and place of interest.

### Haifa Bay area

Haifa Bay is an industrially active area, especially in chemical industry and oil refineries. Based on the 2018 annual summary of monitored pollutants the emissions of few specific pollutants in Haifa Bay are high, even though the ambient concentrations have been slowly decreasing due to national intervention programs implemented in the area [14]. The well-known adverse effect of exposure to particulate matter (PM) and gases of industrial sources has put the Haifa bay area in the center of attention of many scientists, such as environmentalists and epidemiologists in Israel. During the last decade, investigators have reported increased rates of cancer, respiratory illnesses and adverse birth outcome among Haifa residents [15–17] A position paper issued by the Ministry of Health urging to decrease pollution levels in the region and add research at an individual level of residents was published in 2016 [18] . The majority of studies on Haifa Bay have used an ecological or semi-ecological methodology, in which the ambient air pollution is applied as a proxy of the actual exposure. Nevertheless, ambient estimates frequently deviate from the true personal exposure and are confounded by socio-economic status, occupation, smoking, nutrition and water consumption, rendering it hard to account for in a standard analysis, leading to residual confounding and spurious or biased associations.

Alternatively, researchers can apply an HBM methodology. To efficiently estimate an internal exposure of residents to pollutants in the region of 20.5 km^2^ and consequently compare it to the rest of the country, one has to obtain a large amount of biological samples. To this effect we could harness the advantage of samples already been collected routinely, such as during volunteer blood donations organized by Magen David Adom national blood services.

### Magen David Adom

**Fig. 1:**
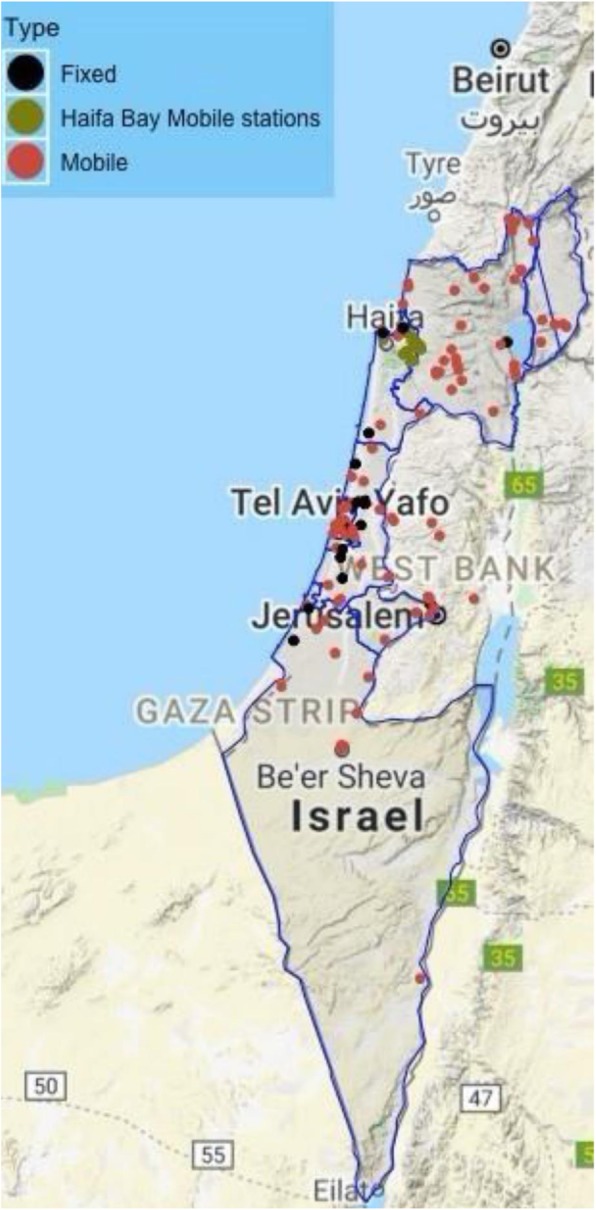
Distribution of collecting units in Haifa Bay and the rest of Israel. The map was produced by the authors using the R software

In Israel, collection of blood donations from volunteer blood donors nationwide is the legal responsibility of Magen David Adom (MDA), a statutory, non for profit organization [19]. In addition, the National Blood Services laboratories of MDA are responsible for the processing, testing and distribution of blood units and components to all the hospitals in the country and to the Israel Defense Forces (IDF) [20]. The national Blood services are managed under the utmost strictest of regulatory and quality assurance standards. MDA donors’ population comprises of 220.000 volunteers per year, 78% being Israeli natives, 75% are 17–40 years old males and 80% are returning donors. Ninety (90%) percent of blood donors are recruited by MDA teams who venture out in schools, factories, community centers and army camps while the other 10% are donated in in fixed-sites donor rooms at MDA stations scattered all over Israel (Fig. 1). MDA collects on a daily basis some 1000 blood units, from all corners of the country.

As a part of this meticulous process, donors are required to fill out an extensive Donor Health Questionnaire (DHQ), which is electronically recorded later. The DHQ contains information regarding their acute and chronic health conditions, including medications, their demographic status and permanent address, and an informed consent form for (a) using the blood donation for medical purposes and (b) a separate informed consent for using the donation for research purposes, in case the donation or parts of it are not required for clinical treatment. As for the HBM purposes we intent to use a small residual blood samples from the test tubes used, the recruitment process will not require additional step in signing an informed consent form of the blood donors. The blood samples used for the study will all have to be screened to be negative for transfusion transmitted diseases (TTD) thus ensuring the safety of handling the samples.

As healthy volunteers, MDA donors represent an ideal population for the precise assessment of the environmental exposure, as they are healthy enough to be eligible for blood donations and are likely to conduct an active life style. In this way, one can view blood donors as human bio-monitors, who can indicate an internal intake of pollution over time without too much confounding factors. Using the blood donors population for the purposes of HBM has been explored in the past on a sample of close to 1000 donors attending a hematherapic collecting unit, whereas donors have been administered a questionnaire and their serum was tested for organochlorine pesticides. Albeit, successfully accomplished, the study was not seen as an attempt of a new HBM routine [21].

Our main objective in the current study is to compare blood samples from donors residents of the Haifa Bay to the rest of the country in terms of their exposure to pollutants, while taking an advantage of a well-established, efficiently working national system of blood collection and processing.

Another byproduct of the study will be in gaining an experience in building a platform for HBM with fine spatial and temporal resolution.

The sampling method of donations used for the study will be aimed to comprise a geographically representative sample of the Haifa Bay area (study area) with the general population of Israel (reference). As MDA permanent and mobile stations are spread all over the country, the population of donors can serve a geographically valid proxy for the study areas.

### Biomarkers indicative of pollution

The modifiable industry or transport-related composites of ambient pollution can be measured by a concentration of organic compounds, as well as dozens of chemical elements, e.g. heavy metals. The latter are also known to present a serious risk to health. Based on International Agency for Research on Cancer (IARC) many of the heavy metals assessed have been defined as carcinogenic to humans [22]. In addition to cancer, heavy metals have been linked to diabetes [23], asthma, hepatotoxicity, obesity, reduced visual acuity [24] and neurological morbidity, e.g. multiple sclerosis [25]. For this reason, knowledge of heavy metals’ concentrations in blood of residents is an important asset in the environmental research. We will focus our study on detection of presence of lead (Pb), cadmium (Cd), arsenic (As) and chromium (Cr) in the study areas.

### Environmental chemicals for testing

Multiple occupational studies have linked between ambient exposure to specific elements at a working place and their concentrations in workers’ biological samples. For instance, a strong correlative link has been shown for Cr, molybdenum (Mo), tungsten (W) and vanadium (V) in blood and urine and for manganese (Mn) in blood [26]. Studies in general population usually attempt linking proximity to industrial zone with residents’ biomarkers. Thus, elevated blood and urine levels of Pb, Cd and mercury (Hg) were found related to plants nearby in Belgium - based on 205 residents [27], in Korea - based on 2020 residents [28], and northern France (Cd and Hg) [29]. In the NHANES in 1988–1994 and 1999–2008, Pb values in blood were related to the Pb air measurements as estimated by 7 monitoring stations [30].

Our team has tested urinary samples from 143 pregnant Arab-Bedouin women for 25 metals. We identified multiple links between metals’ concentrations in urine with environmental factors in pregnant women’s household and outdoor pollution (Karakis I. et al., “How metal concentrations are associated with morbidity, an eight years follow-up among women of childbearing age”). Specifically, exposure to elevated Nitrogen Oxide (NOx), Nitrogen Dioxide (NO_2_) and Nitrogen Monoxide (NO) 3-months prior to testing was associated with higher nickel (Ni), aluminum (Al), Pb and Cr in urine. Women exposed to elevated PM_2.5_ and PM_10_ were more likely to have higher concentrations of Co, Ni, thallium (Tl), Pb, iron (Fe), strontium (Sr), barium (Ba), silver (Ag) and Cr; sulfur dioxide (SO_2_) – to Co and Ni. Complaints on noise was related to higher Co, Ni, Mo, Cd and V in urine by increasing their concentration times 2.42–9.43 (at maximal *p*-value< 0.081), while smell complaints were associated with higher Co and Sr times 2.55–6.27 (maximal p-value< 0.025).

In conclusion, based on research by others as well as our group we are certain that environment will probably have an impact on internal dose of pollutants in the blood.

## Methods

*Study population* will be comprised of healthy, volunteer blood donors from all locations in Israel. We will exclude donations, for which their donor’s residence address is missing or written unclear. Likewise, we will exclude donors at the military bases.

As mentioned previously, donors are requested to sign an informed consent to allow the usage of these samples in the research. All samples will be screened to assure that only those who have signed the wavier will be included in the study.

**Fig. 2:**
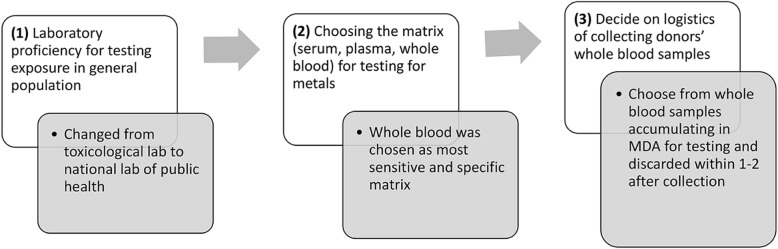
Flowchart of laboratory plan development for metal testing

The study protocol development followed the stages captured in Fig. 2, starting from (1) the laboratory feasibility assessment, followed by (2) choosing the right medium for testing and concluded by (3) adjusting to the Blood Bank routine restraints.

### Laboratory methods for heavy metals assessment

The testing for Pb, Cd, As and Cr by the National Public Health Laboratory. The laboratory is using an Inductively Coupled Plasma Mass Spectrometry (ICP-MS) instrumentation equipped with Integrated Sample Introduction System-Discrete Sampling (ISIS-DS) accessory that enables introducing low sample volume to the device and High Matrix Interface (HMI) technology. These two features help with decreasing system contamination and matrix interference, and allow reaching high sensitivity in testing for metals in general population.

To calibrate the testing procedure for each of the metals we first identified the optimal medium for testing, i.e. serum, plasma and whole blood, which would fit all 4 metals, while all mediums are available at various stages of blood donation processing. The following are the main considerations factoring into our decision of choosing the right medium available at blood bank production.

### Sensitivity

In blood, metals can be present both in the non-cellular fraction (i.e. plasma and serum) and intra-cellular compartment (particularly erythrocytes) [31, 32, 5]. Metals have varying affinity for each compartment, depending on their chemical properties, e.g., Pb is known to have a strong affinity for erythrocytes [33]. Several studies have reported a significant difference between concentrations in whole blood and serum for trace elements and heavy metals. For instance, researchers examining blood samples of 1016 seniors in Sweden found that Al, Cd, Co, Cr, Hg, Mn, Ni, Pb, Zn concentrations were significantly higher in whole blood compared to serum [32].

### Specificity

- From the perspective of storage, the anticoagulation factor had to be taken into account. Serum is the liquid component of the blood remaining once the blood has clotted. Therefore, the serum containers do not require adding co-angulation factors for storage. On the other hand, plasma is the liquid part that remains when clotting is prevented by an added anticoagulant, specifically Ethylenediaminetetraacetic (EDTA). Without EDTA in a collection tube, blood samples will normally clot within a few minutes [34]. Both serum and plasma extraction processes require centrifugation following blood collection.
- To reduce contamination by metals in the laboratory equipment, it was essential to rule out the contaminations introduced by metal needles.
- As the equipment used for collection and storage in the Blood bank may contains metals, we had to account for the leaching potential in the analytical method of testing. Importantly, the sampling tubes are different for the three mediums and therefore had to be assessed separately.

The whole blood matrix with the additive of EDTA, demonstrated the best results in terms of the highest sensitivity and specificity, and was therefore chosen as the optimal medium for testing for the metals planned in the study (Pb, Cd, As and Cr.)

To account for smoking habit of the blood donors (a confounding factor not reported in the questionnaire) we will test a random sample of 80 for Cotinine. As Cotinine is a valid biomarker of smoking, its readings will be compared to their Cd and Pb results in attempt to verify a cut off to be applied to Cd / Pb testing as a proxy for smoking. The final ranges for distinguishing between smokers and non-smokers will be based on the study findings and in correspondence with the ranges of Cd and Pb reported for similar populations.

### Sampling and samples handling

Following the insights received from the laboratory investigation and the availability of the testing materials, the study team adjusted the study logistics of sample collection of whole blood. Whole blood samples in EDTA-containing test-tube will be collected from eligible blood donors, who consented to their use for research purposes. After completing the required screening in MDA blood services laboratories, these test-tubes are normally kept for additional 1–2 days for further testing, if required, and then discarded. After completion of all the needed tests in MDA Blood Services the samples will not be discarded, but assigned to the study. Half of all samples tested will originate from donors in the Haifa Bay.

The sampling matrix will be based on the locations of blood mobile drives and the fixed donor rooms at MDA first-aid stations. This approach assumes that all geographical locations of the blood collecting units correspond to the geographical locations of Israel residents (Fig. 1). To ensure representative sampling of residents in non-Haifa Bay area, the sampling of the controls will be done in strata of the 4 main regions in Israel.

**Fig. 3:**
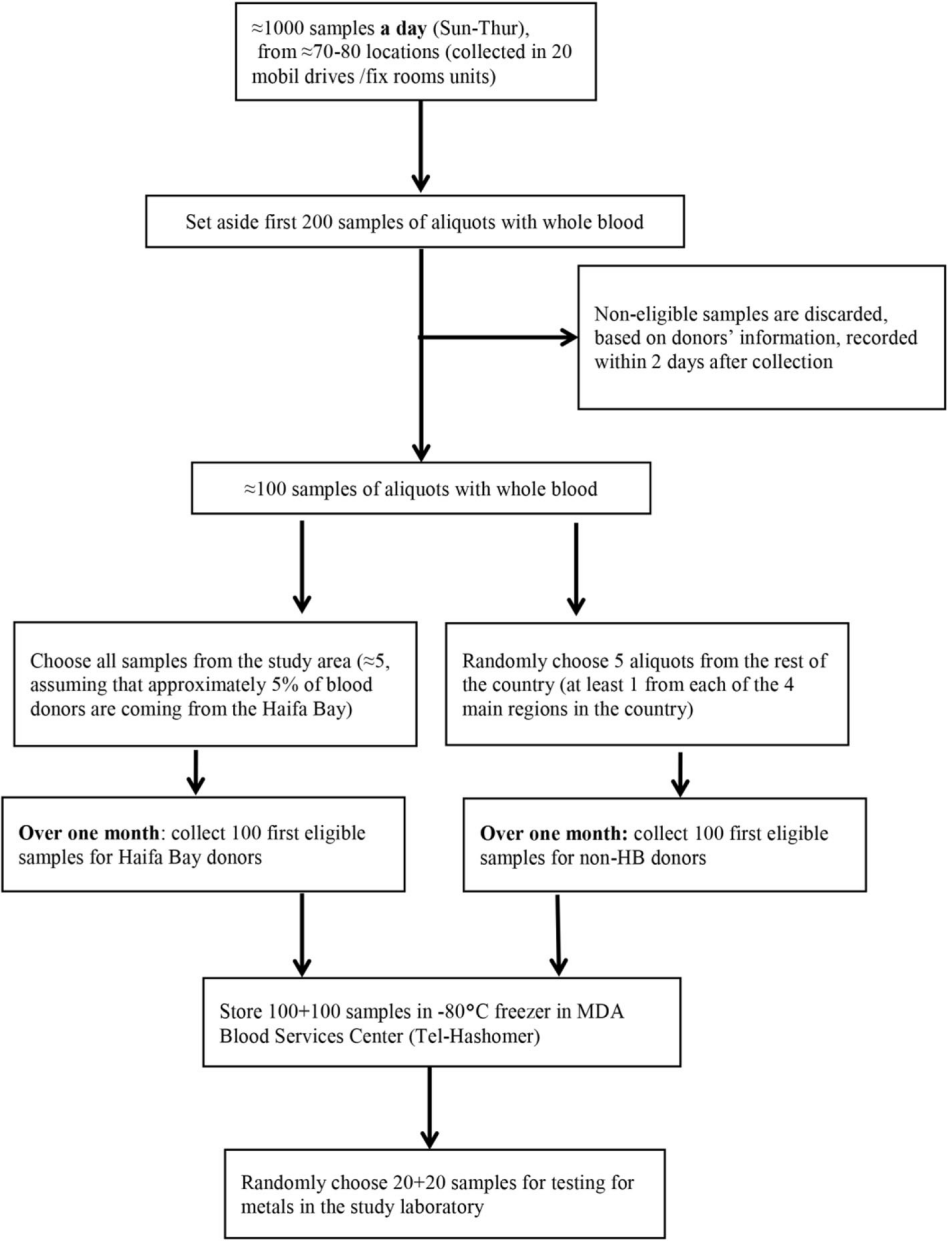
Flowchart of collecting and testing blood samples in the study

Enrollment of a blood donor who agreed to using their test tubes/donation also for research purposes will essentially mean that the regular process for blood donation, processing and distribution plan will not be changed. The sampling algorithm will closely follow the one described in Fig. 3.

The samples assigned to the study will be marked by a survey label and assigned a study number. Each sample will be marked with a unique barcode used by MDA to identify all components associated with each and every donation. Demographic information (e.g. gender, age, zip code of residential address) from the donors’ questionnaire will be collected and assigned for each sample assigned to the study. Batches of 40 samples (half from study area and half controls will be stored in − 80 °C, in one EDTA test tube, 8.5 cc). Once or twice a month the samples will be transferred for testing to the testing laboratory.

### Environmental exposures

We will use blood collection locations for short-term exposures and donors’ zip code of residence for long-term exposures. These locations will be geocoded locally by our group including geocoding validation as commonly done in similar studies and further linked with the environmental exposure database, which will include exposures to PM_10_ and PM_2.5_, NO_2_, sulfate dioxide (SO_2_), ozone (O_3_), carbon monoxide (CO), air temperature (°C) and relative humidity (%).

The exposure to PM_2.5_, PM_10_ and air temperature will be based on hybrid satellite-based exposure models. These models were developed by our team, and will incorporate daily satellite remote sensing data [35–37]. Model performance for particles’ exposure was as high standard as USA, and Europe models, with out-of-sample cross-validation (CV) R^2^ values of 0.84–0.87 for the PM_2.5_ model and 0.90–0.92 for the PM10 model. Likewise, models' goodness-of-fit for temperature was high (CV R^2^ resulting on average at 0.986 and 0.987, for two different satellites) [35]. Data obtained from the satellite-based models are on a daily resolution of 24 h.

Data on relative humidity and precipitation will be obtained from the meteorological stations in Israel. Information on pollutants, i.e. NO_2_, SO_2_, O_3_, CO and relative humidity (%) will be completed from the high-temporal resolution records of the monitoring stations managed by the Ministry of Environmental Protection spread throughout Israel, and available at a daily resolution.

### Data processing

The information collected in the study will include:

1. The time and location of sample’s collection, and donors’ zip code of their permanent address
2. The location parameters will be geocoded, and linked with the ambient exposure at the location and time of collection (short-term exposures). We will also assess exposure throughout 1 year prior to the donation and will link it with the permanent address of a donor (long-term exposures) and a distance of his/her permanent residence from the main roads.
3. The laboratory results on heavy metals blood levels.

The samples’ collection is planned for 24 months in 2020–2021.

### Statistical analysis

At first, we will compare the Haifa Bay donors with the general population of non-Haifa Bay donors, in terms of their main demographic and location characteristics during the study period, using standard statistical methods, i.e., mean ± stdev, median, 95% confidence intervals (CI), Chi-square, t and non-parametric tests. Similarity in distribution will ensure the unbiased comparison of the biomonitoring results. In the opposite case, we will consider individual matching of non-Haifa Bay area donors (study population) with the rest of the donors (reference). The readings below the level of quantity detection will be imputed by a square root of the detection level of the study laboratory, following the standard practice of lab data reporting. The biomonitoring data will be further described by geometric mean, median, minimum, maximum and 95%CI. The description will be provided separately for the subsample of Haifa Bay donors and the rest of Israel, also in strata for main geographical locations, e.g., southern, central, etc. and for the main municipalities in Israel. Additional comparisons will be made of Haifa Bay donors within strata of rural and urban areas, and also by the source of pollution. Biomarker readings by regions will be compared by ratio t-test. The association between ambient environmental exposures and biomarker readings will be analyzed using a log-normal regression, whereas the biomarker is the log-transformed predicted outcome in the regression and ambient exposure is the main independent variable. The analysis will be adjusted to all possible confounders recorded in the database. Prevalence ratio (PR) will represent the main estimate of association at study.

*Sample size considerations:* over the course of 2 years we will collect up to 4800 samples eligible and evaluable for analysis, about 1000 of them will be tested for metals. For a simple comparison of donors in HBA with the rest of the population in terms of proportion of donors in whom at least one metal was detected, and assuming the power of at least 80%, the proportion of metals in general population equal 15% and a two-sided test with significance level of 5%, the sample of 900 will allow to distinguish the minimal difference of 7.5%, i.e. Relative Risk as low as 1.46. Comparison of continuous metals’ levels will yield a higher power.

## Discussion

Few considerations in preparing the current protocol are important to mention.

- In the protocol we tried to maximally adapt to the existing blood banking system and avoid disrupting its processes. This principle led us in many decisions. For instance, the enrollment stage will start only once the samples arrive at the blood bank and not at the mobile units where we could have encouraged the donors to sign their agreement to use their donations in studies. This way we deliberately limit ourselves to donors who did not forget to check the required field in the consent form, however, we do not disrupt the process of blood donation collection.
- Additionally, we aimed to create a methodology that would be maximally simple in its logistics and hence, feasible, for adapting as a routine procedure in future.
- The blood banking system is different in every country and therefore our methods should not be copied directly, but will need to undergo certain adjustments.
- Blood is not an optimal medium for testing some environmental chemicals, in which case the platform of blood donors may not work.

The current study will establish a framework of dynamic national biomonitoring by assessing the exposure to selected chemicals in Israel. Assessments of potentially hazardous elements in donated blood will allow to geographically map areas of hazardous exposure. Besides providing an answer to the main comparison in the study, the information collected in the proposed survey will be at researchers’ disposal for testing new hypotheses in the area and may lead to a new paradigm of tailored environmental preventions measures.

In future, the collected biobank will be able to serve as a platform for more operational questions in public health requiring an expedited answer, whether based on already collected samples or on dynamically (within a matter of days) adjustable process of sampling.

## Data Availability

*Data Statement*: Data will be provided upon request and following the approval by the IRB approval and MDA research committee.

## Abbreviations

CI: Confidence interval
CDC: Center of disease control and prevention
CO: Carbon monoxide
CV: Cross-validation
DHQ: Donor health questionnaire
EDTA: Ethylenediaminetetraacetic
EHMS: Environmental health monitoring system
HBM: Human biomonitoring
HMI: High matrix interface
ICP-MS: Inductively coupled plasma mass spectrometry
IDF: Israel defense forces
MABAT: Ministry of health national health and nutrition survey
MDA: Magen David Adom
NHANES: National health and nutrition examination surveys
NO: Nitrogen monoxide
NO_2_: Nitrogen dioxide
NOx: Nitrogen oxide
O_3_: Ozone
PAH: Polycyclic aromatic hydrocarbon
PCBs: Polychlorinated biphenyls
PM_2.5_: Particulate matter with diameter < 2.5 μm
PM_10_: Particulate matter with diameter < 10 μm
PR: Prevalence ratio
SO_2_: Sulfur dioxide
TTD: Transfusion transmitted diseases
VOCs: Volatile organic compounds
Ag: Silver
Al: Aluminum
As: Arsenic
Ba: Barium
Cd: Cadmium
Co: Cobalt
Cr: Chromium
Fe: Iron
Hg: Mercury
Mn: Manganese
Mo: Molybdenum
Ni: Nickel
Pb: Lead
Sr: Strontium
Tl: Thallium
V: Vanadium
